# Disentangling the effects of sulfate and other seawater ions on microbial communities and greenhouse gas emissions in a coastal forested wetland

**DOI:** 10.1093/ismeco/ycae040

**Published:** 2024-03-26

**Authors:** Clifton P Bueno de Mesquita, Wyatt H Hartman, Marcelo Ardón, Susannah G Tringe

**Affiliations:** Department of Energy Joint Genome Institute, Lawrence Berkeley National Laboratory, Berkeley, CA 94720, United States; Department of Energy Joint Genome Institute, Lawrence Berkeley National Laboratory, Berkeley, CA 94720, United States; Department of Forestry and Environmental Resources, North Carolina State University, Raleigh, NC 27695, United States; Department of Energy Joint Genome Institute, Lawrence Berkeley National Laboratory, Berkeley, CA 94720, United States; Environmental Genomics and Systems Biology Division, Lawrence Berkeley National Laboratory, Berkeley, CA 94720, United States

**Keywords:** soil microbes, wetlands, seawater intrusion, sulfate, methane

## Abstract

Seawater intrusion into freshwater wetlands causes changes in microbial communities and biogeochemistry, but the exact mechanisms driving these changes remain unclear. Here we use a manipulative laboratory microcosm experiment, combined with DNA sequencing and biogeochemical measurements, to tease apart the effects of sulfate from other seawater ions. We examined changes in microbial taxonomy and function as well as emissions of carbon dioxide, methane, and nitrous oxide in response to changes in ion concentrations. Greenhouse gas emissions and microbial richness and composition were altered by artificial seawater regardless of whether sulfate was present, whereas sulfate alone did not alter emissions or communities. Surprisingly, addition of sulfate alone did not lead to increases in the abundance of sulfate reducing bacteria or sulfur cycling genes. Similarly, genes involved in carbon, nitrogen, and phosphorus cycling responded more strongly to artificial seawater than to sulfate. These results suggest that other ions present in seawater, not sulfate, drive ecological and biogeochemical responses to seawater intrusion and may be drivers of increased methane emissions in soils that received artificial seawater addition. A better understanding of how the different components of salt water alter microbial community composition and function is necessary to forecast the consequences of coastal wetland salinization.

## Introduction

Wetlands are highly productive and valuable habitats that store vast quantities of carbon, but are also the largest natural source of the potent greenhouse gas methane (CH_4_) [1]. Understanding the drivers of wetland greenhouse gas emissions is thus important, especially given discussion of the potential for coastal wetlands to sequester carbon in what has been referred to as “blue carbon” [2-5]. Coastal wetlands and other ecosystems such as seagrass meadows have the potential to sequester large quantities of carbon; however, increases in carbon (C) storage could potentially be offset by greenhouse gas emissions [6, 7], which warrants further study especially in the context of global change factors such as sea level rise.

One source of uncertainty for carbon storage in coastal wetlands is how increases in salinity—here defined as the total amount of dissolved salts in water—will affect greenhouse gas emissions, especially given that two principal factors driving coastal salinization—sea level rise and drought—are predicted to increase in the future with climate change [8]. Sea levels have risen 20 cm since 1901 and are currently (2006–18) rising at a global mean rate of 3.7 mm per year [8], which will lead to seawater intrusion into formerly freshwater areas. Droughts have increased in frequency and severity in many parts of the world, which decreases freshwater inputs into estuarine and coastal ecosystems, thereby increasing salinity [8, 9]. Several other anthropogenic factors such as water management and agriculture also contribute to coastal salinization, which, in turn, contributes to several negative consequences for ecosystems including loss of biodiversity and ecosystem services [10].

Theory based on ecology and thermodynamics predicts that methane emissions from coastal systems will decrease with increasing salinity, as the concurrent increase in sulfate from seawater will promote sulfate-reducing organisms that can outcompete methanogens for shared resources in anaerobic respiration (such as acetate and hydrogen) [11-15]. A log-linear decrease in CH_4_ emissions with salinity has been found in several studies and meta-analyses across multiple habitat types including mangroves, salt marshes, and seagrass meadows [16-18]. However, a recent summary of laboratory microcosm experiments testing salinity–methane relationships reported eight negative relationships, two “positive” relationships, and one neutral relationship [19]. This variation in outcomes was attributed in part to hydrological setting, but microbial ecology is another main factor that could contribute to such discrepancies and warrants further research [20, 21]. More specifically, hypotheses about the roles of sulfate, sulfate reduction, and sulfate reducing bacterial populations need to be more explicitly examined. Sulfate reducers encompass diverse organisms that not only compete with methanogens, but can also be syntrophic with methanogens, as certain sulfate reducers can produce acetate and hydrogen which would then fuel methanogenesis from those substrates [22]. Methanogens also encompass a range of taxonomic and functional diversity, as they can perform one or more of four different types of methanogenic pathways (hydrogenotrophic, acetoclastic, methyl-dismutation, and methyl-reduction), which would be differentially affected by sulfate reducers [23]. For example, most methyl-based pathways are likely not affected by competition with sulfate reducers [24-27]. Furthermore, seawater contains many other ions besides sulfate and sodium (Na^+^)—the cations potassium (K^+^), calcium (Ca^+^), magnesium (Mg^2+^), and strontium (Sr^2+^), and the anions chloride (Cl^−^), bromide (Br^−^), and bicarbonate (HCO_3_^−^). While sulfate is the only seawater ion used directly as an electron acceptor for respiration, these other ingredients are involved in other biogeochemical cycles and some act as nutrients for biological growth, and therefore may have effects on microbial communities and greenhouse gas emissions that are separate from those of sulfate.

Previous research across salinity gradients has highlighted the strong role of salinity on microbial taxonomy and function in both the water column and sediments [28-36]. At the global scale, salinity is a primary variable structuring microbial communities [37]. However, it is important to note that there are different types of salinity based on the source of the salts, and this can have different effects on microbial communities [38, 39]. Marine, coastal, and some inland ecosystems are characterized by seawater-derived salinity (i.e. “thalassohaline”) and have similar ionic compositions as seawater. Other inland ecosystems such as soda lakes, certain inland seas such as the Dead Sea, and desert soils contain non-seawater derived salts (i.e. “athalassohaline”), which have their own unique effects on microbial metabolism [40-43]. In this work, we are primarily interested in the seawater-derived salinities across estuaries, which range from more freshwater areas upstream to brackish and polyhaline areas closer to the sea [29, 44]. Work on methanogens found decreases in relative abundance in brackish wetlands compared with freshwater wetlands in the same estuary [45]. The body of work on the effects of salinity on microbes demonstrates the primary role of salinity in structuring microbial communities and has delineated some differences among types of ions. However, one outstanding limitation is a mechanistic explanation of how each seawater component affects microbes and biogeochemical cycling, which is crucial for understanding methane fluxes given the dynamics between sulfate reducers and methanogens.

In this study, we utilized a manipulative laboratory incubation experiment to isolate the effects of sulfate from other seawater ions on microbial and biogeochemical responses when freshwater wetland soils are exposed to simulated seawater intrusion with artificial seawater (ASW) additions. The treatments included controls (+ DI water), + sulfate only (+SO4), +ASW without sulfate (+ASW-SO4), and +ASW (which by default contains sulfate). We hypothesized that both +ASW and +SO4 alone would alter microbial community taxonomy and function, increasing sulfate reducer populations and genes and reducing methanogen populations and genes, and decrease methane emissions, whereas ASW additions without sulfate would have less of an effect on microbial communities and greenhouse gas fluxes and be more similar to controls. Results on soil solution chemistry [46, 47] and greenhouse gas emissions [19] from this experiment have been previously reported. Here we build on our previous work by examining microbial community composition and function, and their connections to greenhouse gas emissions.

## Materials and methods

Soil cores were collected in 2011 from the Timberlake Observatory for Wetland Restoration in North Carolina, USA (35°54′22′′ N, 76°09′25′′ W, 0-m elevation), a forested freshwater wetland in the Alligator River estuary that connects to the Albemarle Sound. The wetland was restored in 2004–06 from prior use as a corn field. The site had not experienced saltwater incursion for at least 20 years prior to sampling. The collection site is characterized by Eutric Histosol soils and Atlantic white cedar vegetation [48, 49]. Laboratory microcosms were started with intact soil cores 2.5 cm in diameter and 30-cm deep, which then received either deionized water (control), artificial seawater (+ASW), artificial seawater without sulfate (+ASW-SO4), and sulfate only (+SO4). Water level was maintained at the soil surface; this was monitored every 2 days and refilled as necessary with the appropriate solution. The water column was not mixed. Another 10 field soil cores (2.5-cm diameter, 30-cm deep) were collected to determine initial field soil characteristics and microbial community composition. Aliquots from these field cores were taken from 0- to 5-cm depth and 10- to 15-cm depth and stored at −20°C for DNA sequencing. ASW composition followed the recipe of Kester *et al*. [50], adjusted to 5-ppt salinity. The recipe (in g/L unless otherwise stated) was as follows: 3.421 NaCl, 0.573 K_2_SO_4_, 0.097 KCl, 0.028 NaHCO_3_, 0.001 KBr, 0.004 H_3_BO_3_, 0.429 mg/L NaF, 8.15 mL MgCl_2_-6H_2_O (1 M), 1.601 mL CaCl_2_-2H_2_O (1 M), 0.130 mL SrCl_2_-6H_2_O (0.1 M). In the ASW-SO4 treatment, the absent K_2_SO_4_ was replaced with an equivalent amount of additional NaCl and KCl. To measure CO_2_, CH_4_, and N_2_O, cores were fitted with a gas tight lid with a Swagelok brass sampling port with a rubber septum (0.6 cm). Headspace gas samples were collected immediately and after 1 h into evacuated 8-ml gas vials and gasses were quantified on a Shimadzu 17A gas chromatograph with an electron capture detector, flame ionization detector, and methanizer (Shimadzu Scientific Instruments, Columbia, MD, USA). The experiment proceeded for 12 weeks, after which soils from 0- to 5-cm depth and 10- to 15-cm depth profiles were collected, a portion stored at −20°C for DNA sequencing, and another aliquot analyzed for porewater and soil biogeochemistry. Measured variables were salinity (defined as Cl^−^ concentration in ppt), bromide (Br^−^), porewater nitrate (NO_3_^−^), sulfate (SO_4_^2^)^−^, total organic carbon (TOC), dissolved organic nitrogen (DON), dissolved inorganic nitrogen (DIN), total nitrogen (TN), ammonium (NH_4_^+^), phosphate (PO_4_^3−^), and soil pH, % carbon (C), and % nitrogen (N). C:N was calculated from the % C and % N data. Furthermore, at Day 14 of the experiment, oxygen and hydrogen sulfide were measured in the top 5 cm of water with microelectrode probes (Bernot Laboratory, Ball State University) [51]. Detailed descriptions of the methodology have been provided in previous publications [46].

### Microbial sequencing and analysis

Soils were frozen at −20°C until DNA extraction and sequencing. DNA was extracted from 0.3 g of soil with a Qiagen DNeasy PowerSoil kit following the manufacturer’s instructions. A subset of DNA was sent for shotgun metagenomic sequencing, whereas another subset was used for PCR amplification of the V4 region of the 16S rRNA gene, following the standard methods of the US Department of Energy Joint Genome Institute (JGI) [52]. Shotgun DNA was sequenced on a HiSeq 2000 (Illumina Inc., CA, USA), whereas amplicon DNA was sequenced on an Illumina MiSeq 2000 with paired-end 150 base pair chemistry. All sequencing was performed at the JGI.

Raw metagenomic data were assembled with MEGAhit [53] and then uploaded to the JGI’s IMG/M database to undergo their standard taxonomic and functional annotation pipeline [54]. Counts of KEGG orthology (KO) groups were downloaded using the Statistical Analysis tool on IMG/M. Counts were normalized with DESeq2 [55] and converted to counts per million. Metagenome-assembled genomes (MAGs) were created by binning contigs in each metagenome (mTAG) with CONCOCT [56], MetaBAT [57], and MaxBin2 [58], and then using dRep [59] to dereplicate the MAGs into a set representing unique organisms. dRep uses average nucleotide identity to identify MAGs representing the same organism, and then uses contamination, completeness, strain heterogeneity, and N50 to select the highest quality MAG. Next, checkM [60] was used to select high quality MAGs with over 90% completeness and <5% contamination, based on single-copy marker genes. MAGs were classified taxonomically with GTDB-tk [61] and a maximum likelihood phylogenetic tree was built with a concatenated alignment of 49 clusters of orthologous genes. These analyses were performed on the KBase online platform and are publicly available in narrative ID 138721 [62, 63]. Furthermore, MAGs were annotated with BLASTkoala [64]. MAG abundance in each mTAG was calculated with coverM [65] as the mean coverage of reads mapped to the MAG in each sample. To complement the 16S marker gene analysis and check for consistency among the two methods, we used mTAGs [66] to extract 16S sequences from metagenomes and annotate them taxonomically with the SILVA 138.1 database [67]. This release generally, but not always, follows the taxonomy proposed by the Genome Taxonomy Database (GTDB) [68], but is still beneficial for 16S taxonomic assignment because 16S genes are often missing from MAGs in GTDB [69].

Raw 16S amplicon sequencing data were processed with the iTagger pipeline to quality-filter reads, dereplicate sequences, and cluster sequences into operational taxonomic units (OTUs) at 97% sequence similarity [52]. Taxonomy was assigned using the “assignTaxonomy” function in “dada2” [70], with the SILVA 138.1 database [67]. “mctoolsr” [71] was used to remove chloroplast and mitochondrial DNA, any taxa not assigned at least to the bacterial or archaeal domains, and singletons and doubletons. Following filtering, sequencing depth was 131 845 ± 3373 SE sequences per sample. We used known taxonomy–function relationships to assign functional guilds of interest for anaerobic biogeochemistry [72], using the “Get_16S_guilds_alt” function in a publicly available custom R script “AssignGuilds.R” found in the repository available on Zenodo (https://zenodo.org/doi/10.5281/zenodo.10011195). Data for taxonomic abundance analyses were rarefied to 81 000 sequences per sample, enough to capture the diversity of microbes in each sample while retaining all samples. Data for compositional analysis were not rarefied, but were rather center log ratio transformed [73] with “Zcompositions” [74].

### Statistical analysis

All analyses were performed with R version 4.0 [75]. Metagenomic gene abundances based on KO were normalized via variance stabilizing transformation with the “DESeq2” R package [55]. Normalized KO abundances were tested for differential abundances using both Wald’s tests and likelihood ratio tests, implemented in “DESeq2.” We focused on the responses of a list of 542 genes involved in carbon (including methane), nitrogen, phosphorus, and sulfur cycling (Table S1). The number of OTUs per sample and Shannon diversity were used as microbial alpha-diversity metrics. The effects of treatment and depth (surface 0–5-cm range versus deeper 5–15-cm range) on alpha-diversity were tested with a two-way ANOVA with treatment and depth as fixed effects, followed by Tukey’s post hoc. The interaction term was initially tested and was not significant so the model was run without the interaction term. Significance was tested with Type II sum of squares in “car” [76]. To calculate predictor variable effect size (ω^2^), a separate three-way ANOVA with sulfate, ASW (without sulfate), and depth as factors was performed. The same procedure was performed for greenhouse gas emissions, except without depth, as those measurements were at the entire core level. Residuals were normally distributed (Shapiro–Wilk test, *P* > .05) and variance was homogenous (Levene’s test, *P* > .05). Microbial community composition was assessed by calculating an Aitchison’s distance matrix, which is preferred for compositional data [73], with “compositions” [77] and performing a PERMANOVA to test for effects of treatment, depth, and their interaction, implemented with the “adonis2” function in “vegan” [78]. Pairwise PERMANOVAs between treatments were implemented with “pairwiseAdonis” [79]. Within-group multivariate homogeneity of dispersion was tested with PERMDISP implemented with the “betadisper” function in “vegan.” To compare a presence/absence-based metric with the Aitchison’s metric, we calculated Jaccard dissimilarity with “vegan.” Compositional analysis was done for both 16S iTag marker gene sequence data and mTAG 16S metagenomic-extracted data. Unique and overlapping taxa in the field and the lab were calculated with “mctoolsr.” Effects of environmental variables were tested with distance-based redundancy analysis, implemented with the “capscale” function in “vegan.” Communities were visualized with principal components analysis (PCA), with environmental vectors fit with the “envfit” function in “vegan.” Pearson correlations between CH_4_ flux and chemical variables and certain microbial guild or taxa abundances, and normalized KO gene counts were calculated and *P*-values corrected with false discovery rate. All figures were made with either the “ggplot2” [80] or “pheatmap” [81].

## Results

**Figure 1:**
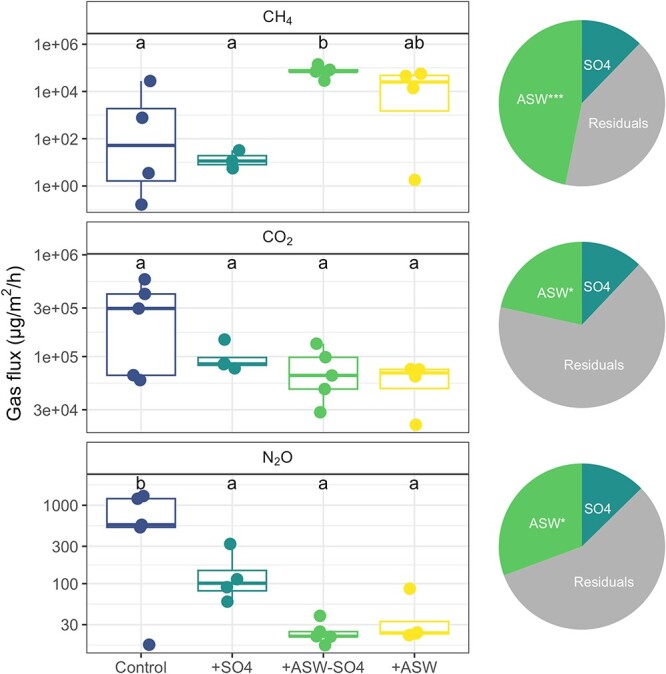
Fluxes of methane (CH_4_), carbon dioxide (CO_2_), and nitrous oxide (N_2_O) in the same soils that were sequenced for microbial community analyses (Week 12 of the experiment). Pies show the effect sizes (ω^2^) for the laboratory treatments, where controls have no sulfate or ASW, +SO4 and + ASW treatments have sulfate, and +ASW-SO4 and + ASW treatments have ASW. “ASW” in the pies refers to the effect of the non-sulfate ASW ions. In the pies, ^*^^*^^*^ = *P* < .001, ^*^^*^ = *P* < .01, ^*^ = *P* < .05 from ANOVA.

CH_4_ fluxes were significantly greater in soils amended with ASW lacking sulfate compared with controls and sulfate-amended soils (Fig. 1), whereas +ASW treatments were intermediate (Fig. 1). CO_2_ fluxes trended in the opposite direction but were not significantly different among the treatments. N_2_O fluxes were significantly greater in controls than all other treatments (Tukey *P* < .05), and were significantly negatively correlated with CH_4_ flux (*R*^2^ = 0.67, *P* < .001) (Fig. 1). In all cases where effects were seen, non-sulfate ASW ions had a significant effect on emissions, whereas sulfate did not, and non-sulfate ASW ions had larger effect sizes than sulfate. In terms of other biogeochemical measurements, both the +ASW and +ASW-SO4 treatments had significantly higher salinity and bromide, whereas the +SO4 and +ASW treatments had greater sulfate concentrations, confirming successful treatment effects (Fig. S1). Typical of freshwater wetlands, the sulfate concentrations measured in porewater in the control samples were low, ranging from 0.23 to 24.96 mg/L at 0–5-cm depth and 0.22 to 18.65 mg/L at 10–15-cm depth (Fig. S1). Other treatment effects on biogeochemistry included greater NH_4_^+^ and DIN concentrations and more basic pH in +ASW and +ASW-SO4 treatments (Fig. S1). There were also significantly greater phosphate concentrations in +ASW-SO4 compared with controls. TOC, DON, TN, NO_3_, %C, %N, and C:N were unaffected by treatment (Fig. S1).

**Figure 2:**
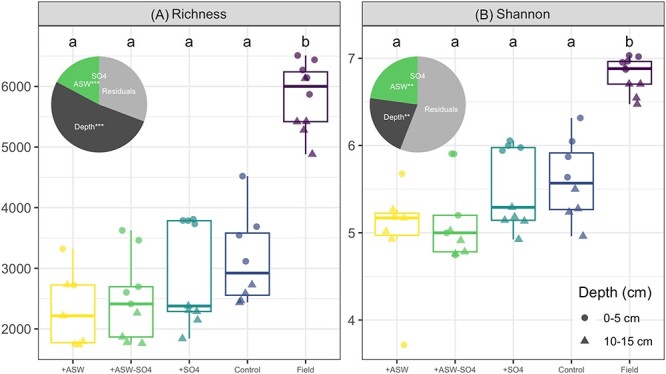
Alpha diversity of 16S rRNA gene amplicon sequences, showing (A) OTU richness and (B) Shannon diversity. Pie insets show the effect sizes (ω^2^) among the laboratory additions, based on just the laboratory data. In the pies, ^*^^*^^*^ = *P* < .001, ^*^^*^ = *P* < .01, ^*^ = *P* < .05 from ANOVA. The *x*-axis is ordered by increasing mean richness.

Microbial alpha diversity (OTU richness and Shannon diversity) was significantly greater in the initial field samples than in the experimental cores, but there were no differences in alpha diversity in pairwise comparisons of the laboratory treatments (Fig. 2). The number of OTUs observed per sample ranged from 1738 to 4520 in the lab, and from 4881 to 6510 in the field. 7721 OTUs (55%) were shared in field and lab samples, whereas 4002 (29%) were found only in the field and 2264 (16%) were found only in the laboratory-incubated soils (Fig. S2). There was a significant effect of depth in all treatments, with greater alpha diversity in the 0–5-cm portion of the core compared with the 5–15-cm deep portion (Fig. 2, Table 1). There was a significant negative effect of non-sulfate ASW ions on richness and Shannon diversity, and no effect of sulfate. Depth had a larger effect size than non-sulfate ASW ions (Fig. 2). Results using 16S rRNA marker gene sequencing (iTags) and 16S rRNA genes extracted from mTAGs were in agreement (Fig. S3).

**Table 1:** Statistical results. ANOVAs were assessed with Type II sum of squares. PERMANOVAs were performed with 999 permutations on Aitchison distances, and included an interaction term after visual inspection of the data. *F* values for PERMANOVAs are pseudo-*F* values computed through permutations.

**Test**	**Dep. var.**	**Ind. var.**	***F***	***R*** ^**2**^	**ω** ^**2**^	***P***
One-way ANOVA	CH_4_ flux	Treatment	9.2			.001
Two-way ANOVA	CH_4_ flux	Non-sulfate ASW	17.2		0.47	<.001
		Sulfate	4.5		0.12	.051
		Residuals			0.41	
One-way ANOVA	CO_2_ flux	Treatment	3.4			.048
Two-way ANOVA	CO_2_ flux	Non-sulfate ASW	4.9		0.22	.043
		Sulfate	2.7		0.12	.119
		Residuals			0.66	
One-way ANOVA	N_2_O flux	Treatment	6.3			.006
Two-way ANOVA	N_2_O flux	Non-sulfate ASW	8.1		0.31	.012
		Sulfate	3.4		0.13	.087
		Residuals			0.57	
Two-way ANOVA	OTU Richness	Treatment	102.3			<.001
		Depth	60.9			<.001
Three-way ANOVA	OTU Richness	Non-sulfate ASW	16.3		0	<.001
		Sulfate	0.47		0.17	.5
		Depth	49.6		0.52	<.001
		Residuals			0.31	
Two-way ANOVA	Shannon	Treatment	33.2			<.001
		Depth	15			<.001
Three-way ANOVA	Shannon	Non-sulfate ASW	11.85		0.23	.002
		Sulfate	0.2		0	.63
		Depth	11.3		0.21	.002
		Residuals			0.56	
PERMANOVA	OTU composition	Treatment	9.5	0.43		.001
		Depth	8.5	0.1		.001
		Interaction	2.1	0.1		.005
PERMANOVA	OTU composition	Non-sulfate ASW	8.1	0.17	0.16	.001
		Sulfate	1.3	0.03	0.02	.195
		Depth	10.1	0.21	0.2	.001
		Residuals		0.6	0.6	

**Figure 3:**
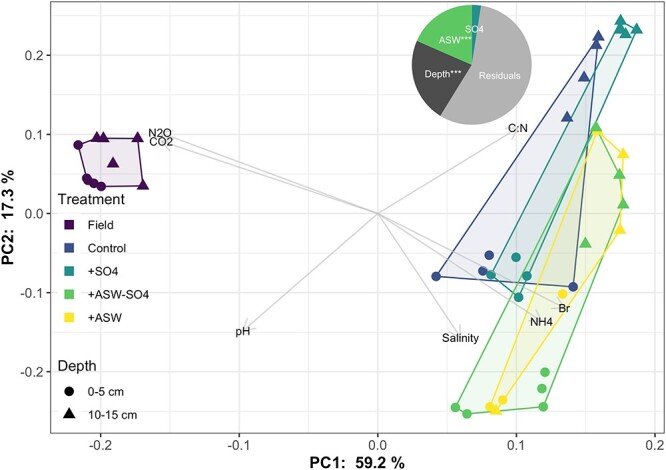
PCA of Aitchison distance calculated from 16S rRNA gene amplicon sequencing data. Vectors show environmental relationships with composition as calculated by “envfit.” Note that CO_2_ and N_2_O were not measured in field samples and those vectors represent higher values in controls and + SO4 samples. Pie inset shows the effect sizes (ω^2^) among the laboratory additions, based on just the laboratory data. In the pie, ^*^^*^^*^ = *P* < .001 from ANOVA.

**Figure 4:**
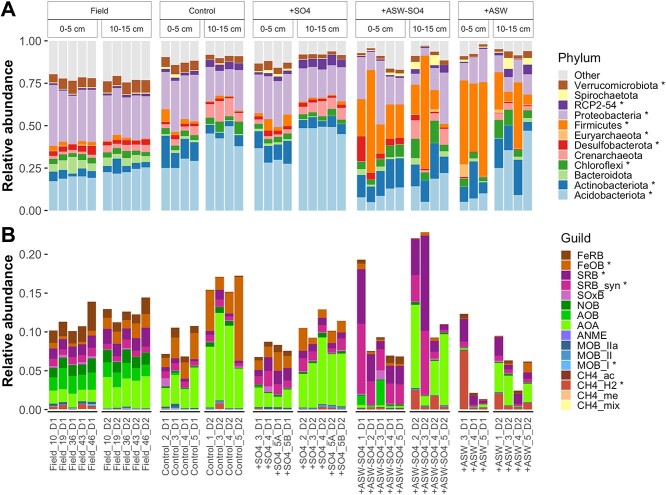
Relative abundance of the top 12 phyla (A) and the 16 functional guilds assigned using known taxonomy–function associations (B) in the 16S rRNA gene amplicon sequencing data. ^*^significant effect of treatment on relative abundance.

Microbial beta diversity was significantly affected by treatment and depth (Table 1, Fig. 3). There was also a significant interaction, such that the effect of depth was greater in the laboratory than in the field. Microbial community composition at the OTU level in field samples was significantly different from all lab treatments, although relative abundances of major phyla and guilds were similar between field samples and lab control samples (Fig. 4). OTU-level composition in laboratory controls and + SO4 treatments were not significantly different from each other, nor were + ASW and + ASW-SO4 treatments. +ASW and + ASW-SO4 treatments were significantly different than controls and + SO4 treatments in OTU-level composition (Pairwise PERMANOVAs, Fig. 3). Variance was similar in all treatments (PERMDISP, *P* > .05). Depth and non-sulfate ASW ions had similar effect sizes, whereas sulfate did not significantly affect composition (Fig. 3). Results were similar whether using Aitchison’s distance (Fig. 3) or Jaccard dissimilarity metrics (Fig. S4). Results using 16S marker gene sequencing and 16S genes extracted from mTAGs were consistent (Fig. S5).

Salinity, ammonium, and bromide were associated with communities in the +ASW treatments, whereas CO_2_ and N_2_O fluxes were correlated with communities in control and + SO4 only treatments; higher C:N ratios were associated with communities from 10- to 15-cm depth, whereas higher pH was associated with communities from 0- to 5-cm depth (envfit, *P* < .05, Fig. 3). The best combination of environmental predictors of community composition was DIN (RDA, *F* = 4.8, *P* = .01), Br (RDA, *F* = 2.4, *P* = .01), and C:N (RDA, *F* = 2.2, *P* = .015).

**Figure 5:**
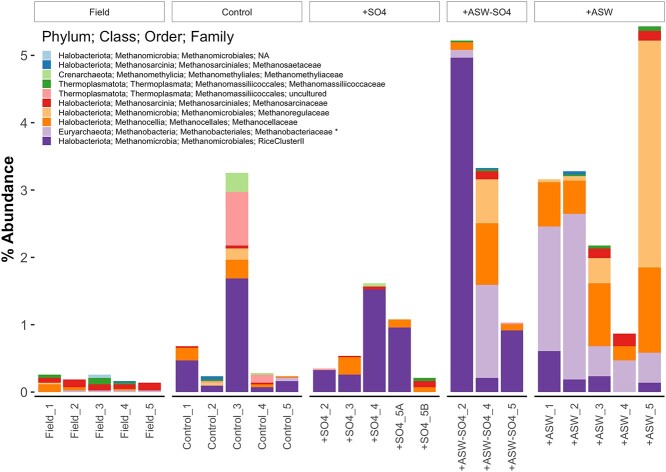
Methanogen abundances from mTAG-derived 16S rRNA gene reads (mTAGs), for 5–15-cm depth. ^*^significant effect of treatment on relative abundance.

Across the whole data set, microbial communities were dominated by the phyla Acidobacteriota, Proteobacteriota, Firmicutes, Actinobacteriota, and Chloroflexi. At the phylum level, the most striking difference was a relative increase in Firmicutes and a relative decrease in Acidobacteriota in the +ASW and + ASW-SO4 treatments (Fig. 4A). Several functional guilds were significantly more abundant in the +ASW treatments, including methanogens (Fig. 4B). There were 10 methanogen families according to both iTags and mTAGs. Five were hydrogenotrophs, one was acetoclastic, two were methyl-reducing, one was methylotrophic, and one was mixotrophic (Methanosarcinaceae) (Fig. 5). The most abundant methanogens were the hydrogenotrophs Methanomicrobiales, Methanobacteriaceae, Methanocellaceae, and Methanoregulaceae. There was no significant increase in the methyl-based methanogens present in our data set (methyl-reducing Methanomassiliicoccaceae or mixotrophic Methanosarcinaceae), which can avoid competition with sulfate reducers. Some archaeal phyla, including methanogen-containing Halobacteriota and Thermoplasmatota phyla, were more abundant in mTAG data than iTag data (Fig. S6), likely because of primer bias. There were 42 genera of methanotrophs (iTags), which were mostly aerobic bacteria; there were very few anaerobic methane oxidizing archaea (Fig. S7). The relative abundance of methane oxidizing taxa was largely unaffected by treatment with the exception of class I methane oxidizing bacteria as a whole, and the genera *Methylomonas* and *Methylocapsa*, which decreased in +ASW and + ASW-SO4 treatments (Figs 4 and S7). As for potential links between methane and nitrogen cycles [82], neither methanotroph abundance nor the ratio of methanogens to methanotrophs was significantly related to the ratio of ammonia oxidizers to nitrite oxidizing bacteria (Fig. S8).

A small set of OTUs from diverse taxa, including Actinobacteriota, Crenarchaeota, Firmicutes, Proteobacteria, Campylobacterota, Desulfobacterota, and Spirochaetota, were the primary drivers of compositional differences among treatments (Fig. S9). These included known ammonia oxidizing archaea, iron oxidizing bacteria, and sulfate reducing bacteria (including syntrophs). The top 10 taxa driving differences explained a cumulative 17%–27% of the compositional difference and ranged from 2% to 60% relative abundance in a sample (mean = 14.2 ± 0.53) (Fig. S9).

**Figure 6:**
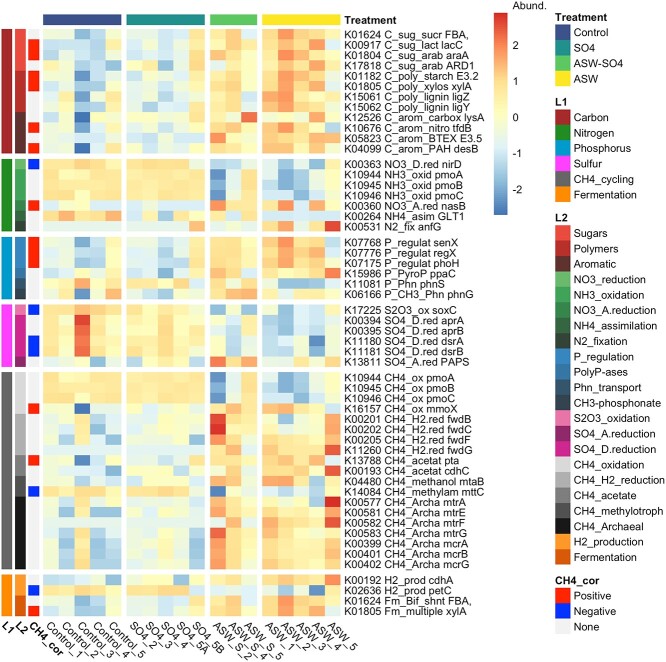
Abundances of KOs involved in CNPS and methane cycling that were significantly affected by treatment. L1 represents a high-level categorization of the genes, whereas L2 represents more specific processes that the genes are involved in. Also shown are correlations with methane flux (“CH4_cor,” left).

There were 50 carbon, nitrogen, phosphorus, and sulfur (CNPS) genes that were differentially abundant in at least one pairwise comparison among experimental treatments, and 19 of these were significantly correlated with methane (13 positive, 6 negative) (Fig. 6). Several sugar, polymer, and aromatic compound degradation genes were positively correlated with methane emissions and more abundant in +ASW (with and without sulfate) treatments. These included *lacC*, *araA*, *xylA*, *tfdB*, and *desB*. Nitrate reduction gene *nirD* as well as ammonium assimilation gene *GLT1* were more abundant in controls and + SO4 treatments. Phosphorus regulation genes *senX*, *regX*, and *phoH* were more abundant in +ASW (with and without sulfate) treatments. Sulfate oxidation gene *soxC* as well as sulfate reduction genes *aprAB* and *dsrAB* were more abundant in both controls and + SO4 treatments. Methane oxidation genes *pmoABC* were more abundant in controls and + SO4 treatments, whereas various methanogenesis genes from all four pathways were more abundant in +ASW-SO4 and + ASW treatments. Lastly, hydrogen production genes had mixed responses, with *cdhA* higher in +ASW treatments but *petC* higher in controls and + SO4 treatments.

**Figure 7:**
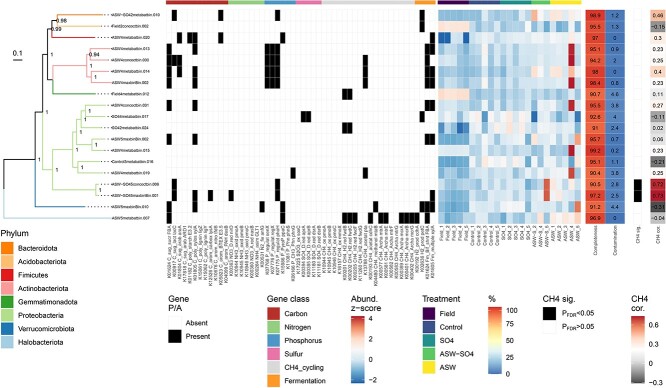
Information on 19 high-quality MAGs recovered from metagenomes, including (from left to right) phylogenetic tree, presence and absence of CNPS and methane genes (same as in Fig. 6), abundance, completeness, contamination, methane correlation significance, and methane correlation coefficient.

There was one high-quality methanogenic MAG recovered, which was classified to the hydrogenotrophic genus *Methanoregula*. This MAG tended to be more abundant in +ASW treatments, in agreement with Methanoregulaceae abundances from 16S data (Fig. 7). However, the MAG was not correlated with CH_4_ fluxes. Two Proteobacterial MAGs were significantly correlated with CH_4_ flux, and these two MAGs contained some of the genes for C and P cycling that were significantly different among the treatments. Most of the high-quality MAGs (13 of 19) contained at least one gene involved in fermentation that was significantly affected by treatment (*cdhA*, *petC*, *fbaA*, *xylA*) (Fig. 7).

## Discussion

Saltwater intrusion into freshwater wetlands will become increasingly widespread and severe with climate change and will have many ramifications for wetland plant, animal, and microbial communities, with important implications for biogeochemical export (e.g. of nitrogen) and greenhouse gas emissions [10, 19, 83]. Sulfate is often cited as a key regulator of saltwater intrusion effects on methane emissions [14, 15, 18, 24, 84], but its role has not been explicitly tested. Here we isolated the effect of sulfate from other seawater ions on microbial communities and greenhouse gas emissions with a manipulative laboratory incubation. Our results demonstrate that for almost every response variable measured, non-sulfate seawater components rather than sulfate drive the microbial and biogeochemical responses. This is demonstrated both by effect size calculations as well as results showing that +ASW treatments, with and without sulfate, were similar, whereas the +SO4 only treatment and controls were more similar to each other. Importantly, the results are not consistent with a lack of effect or unintended effects of the treatments. The treatments worked as designed, as porewater measurements demonstrate elevated sulfate levels in the two treatments in which it was added (+SO4 and + ASW). Rather, despite this observed increase in sulfate concentrations, sulfate reducing bacteria did not increase in relative abundance, and sulfate reduction genes did not increase relative to controls (Figs 4 and 6). However, metagenomic and 16S data are limited in that they only provide relative abundance data. The single measurement of hydrogen sulfide concentrations on Day 14 of the experiment showed increased hydrogen sulfide production in the +SO4 treatment but only a slight increase in the +ASW (also containing sulfate) treatment (Fig. S10A), suggesting potential sensitivity of the resident sulfate reducer populations to salinity.

While here we only present experimental results from Week 12 of the experiment, when soils were collected for microbial sequencing, they agree with the results from the entire time series of the experiment [19]. CH_4_ emissions in the +ASW and + ASW-SO4 treatments compared with the control and + SO4 treatments were increased by Day 21 of the experiment and consistently higher over the following seven sampling timepoints (every 1–2 weeks) until the end of the experiment [19]. While this result is in disagreement with general predictions and previous reviews and metanalyses, it is not alone—CH_4_ fluxes peaked at oligohaline salinities rather than freshwater salinities in the San Francisco Bay/Sacramento-San Joaquin River Delta [72], and ASW additions increased methane emissions in a similar experiment with soils from tidal freshwater wetlands from the Delaware River estuary [85]. Interestingly, microbial composition in controls and + ASW treatments was substantially different both here (Fig. 3) and in the Delaware River, but in both cases, unique suites of methanogenic taxa appeared to shift in relative abundance with ASW addition [86]. Other key results from the full time course of the experiment include increased nitrogen export [46] and decreased dissolved organic carbon export [47], highlighting other biogeochemical changes that occur with ASW addition that may interact with microbial activity and greenhouse gas emissions.

The increased CH_4_ emissions observed in our data are likely driven by a combination of increased methanogenic activity of salt-adapted methanogens, increased methanogenic substrate generation by Firmicutes, decreased abundance and activity of denitrifiers, and lack of sulfate-mediated anaerobic methane oxidation [87], as the relative abundance of ANME archaea was very low. Addition of sulfate (in +SO4 and + ASW treatments) would be most likely to decrease CH_4_ emissions if sulfate reducing bacteria populations outcompeted methanogens and increased in relative abundance. However, in our experiment, sulfate reducing bacteria did not increase in relative abundance and therefore likely did not exert any control over the hydrogenotrophic methanogens (Methanobacteriaceae) that increased in relative abundance with +ASW (and + ASW-SO4). Another possibility is that dissolved oxygen levels decreased with salinity, and since the waters were not thoroughly mixed, the +ASW and + ASW-SO4 treatments became more anaerobic which fueled increased methanogenesis. Oxygen measurements taken on Day 14 of the experiment do not support this hypothesis, as oxygen levels were actually slightly higher in the +ASW treatment, and all treatments were anaerobic at 2-cm depth and below (Fig. S10B).

When seawater is added to a freshwater microbial community (aquatic), the environmental conditions change such that they are more similar to the sea environment, and consequently, microbial communities undergo selection and become more similar to marine microbial communities [88]. However, this process likely takes several years to decades; one year following transplantation of soil cores from a freshwater marsh to a mesohaline marsh, soil microbial communities were unique in composition, resembling neither the transplant origin site community nor the transplant destination site community composition [21]. In our laboratory incubations, all of the laboratory samples were significantly different in microbial composition from the starting field soils after 12 weeks (Fig. 3). The +ASW (with and without sulfate) cores were even more dissimilar to the field than the control or sulfate only treatments, and this was associated with lower taxonomic richness (Fig. 2). This is likely because of the inhibitory effects of dissolved NaCl on many freshwater-adapted taxa; for example, one experiment that used sterile seawater additions, isolating the direct abiotic effect over any indirect biotic effect (e.g. competition), caused a 79% decline in freshwater taxa [88].

Other seawater components besides sulfate include the cations sodium (Na^+^) potassium (K^+^), calcium (Ca^+^), magnesium (Mg^2+^), and strontium (Sr^2+^), and the anions chloride (Cl^−^), bromide (Br^−^), and bicarbonate (HCO_3_^−^). These components appear to enrich certain methanogens such as Methanobacteriaceae (Fig. 5). These results disagree with findings that methanogens were more abundant in freshwater compared with brackish marshes in the same estuary in China [45], and in a tidal freshwater compared with tidal mesohaline marsh in Virginia, USA [21]. However, despite lower abundances of total methanogens, the Methanomicrobiales order did increase following increased salinity, similar to what we observed in our experiment [21]. Both +ASW and + ASW-SO4 treatments enriched taxa with complex carbon degradation genes (e.g. Firmicutes, Actinobacteriota) that are important for initiating carbon degradation, which ultimately provides substrates for methane production. Interestingly, CO_2_ production was not coupled with CH_4_ production, as might be expected as greater overall carbon degradation rates would lead to greater production of both gases, and acetoclastic and methyl-based methanogenesis produce both CH_4_ and CO_2_. CO_2_ emissions decreased with ASW additions and were not affected by sulfate (Fig. 1).

To our knowledge, a decrease in CO_2_ but increase in CH_4_ in response to +ASW has not been previously observed in tidal freshwater wetlands. In a review of carbon mineralization responses to field manipulations to oligohaline, mesohaline, or seawater salinities in several wetland types (forested freshwater, tidal freshwater marsh, brackish peatland), CO_2_ production increased four times, decreased six times, and did not change three times, whereas CH_4_ production was never found to increase, decreased 12 times, and was unaffected three times [89]. CO_2_ and CH_4_ emissions had the same result (i.e. both positively responded or both negatively responded) eight times and contrasting results five times (increased CO_2_ and decreased or no change in CH_4_) [89]. In the aforementioned laboratory study from the Delaware River estuary, both CH_4_ and CO_2_ increased with +ASW. Notably, our field site is not a tidal wetland—it is a forested freshwater wetland, which have been less studied. The distinct biogeochemistry (low pH) and broader microbial community differences could contribute to our unique results compared with tidal freshwater wetlands [86]. Indeed, previous work in three different wetland types—cypress dome, bayhead swamp, and mineral marsh—found that soils in the different wetlands responded differently to simulated seawater intrusion in terms of nutrient export and greenhouse gas production [90]. In particular, the concentration of humic and phenolic compounds, which was not quantified here or in most other studies, could affect how greenhouse gas production responds to seawater intrusion, but this hypothesis remains to be tested. SUVA_254_, a common metric of aromaticity and humics in water, was negatively correlated with CH_4_ flux at our site [19].

The CO_2_:CH_4_ ratio depends on several factors including oxygen concentrations, electron acceptor abundances, nutrient availability, hydrogenation of organic matter, and methanogenesis pathway [91, 92]. In both acetoclastic and methylotrophic methanogenesis, both CO_2_ and CH_4_ are produced [93], but this represents only a small fraction of the total CO_2_ being produced by the microbial community (e.g. from aerobic respiration). However, even in anaerobic environments, CO_2_:CH_4_ production ratios are typically >1, possibly because of hydrogenation of organic matter [91]. Furthermore, greater CH_4_ production was associated with Firmicutes and Proteobacteria fermenters, whereas greater CO_2_ production was associated with a diverse suite of cellobiose-carbon degraders [92]. This partially agrees with our results that show an association between ASW, Firmicutes, and CH_4_ emissions.

A key surprising result is that sulfate reducers were not enriched (16S relative abundance) by treatments containing sulfate. Across individual samples, sulfate reducing bacteria (SRB + SRB_syn) ranged from 0.4% to 21% of the community. Among treatments, relative abundances were highest in the +ASW-SO4 treatment, intermediate in +ASW and + SO4 treatments, and lowest in controls (Fig. 4). These results contrast with observed increases in sulfate reducer abundances following transplantation of tidal freshwater marsh soils to a tidal mesohaline marsh [21]. On the other hand, sulfate reduction genes in metagenomes were most abundant in control and + SO4 treatments and least abundant in +ASW and + ASW-SO4 treatments (Fig. 6). The discrepancy between 16S and metagenomic results in this case could potentially be because of the presence of *dsr* genes in taxa other than sulfate reducing bacteria [94-96]. The lack of difference between control and + SO4, and between +ASW and + ASW-SO4, demonstrates a lack of effect of sulfate on sulfate reduction gene abundances, and rather, an effect of non-sulfate seawater ions. More research is needed on how non-sulfate seawater ions affect the growth and activity of sulfate reducing bacterial taxa, and how a lack of these ions may limit sulfate reduction. Sulfate reducing bacteria are a diverse functional guild with a wide range of salt tolerance and salt optima. For example, SRB from soda lakes can tolerate up to 3 M (175.32 g l^−1^) NaCl, and have an optimum growth rate at around 0.5 M (24.22 g l^−1^) NaCl [97]. On the other hand, an SRB isolated from a freshwater lake grew very slowly at 5 g l^−1^ NaCl and had optimum growth at 1 g l^−1^ NaCl [98]. pH is also an important factor for sulfate reducing bacteria; their lack of response to increased sulfate concentration may be partially explained by their slower growth rates at low pH, even if they make up 2.5% of the community on average (Fig. 4) in field samples with pH < 5 [99]. pH was <5.5 in all laboratory samples and significantly higher in the +ASW treatment compared with the control and + SO4 treatments (Fig. S1). While sulfate reducing bacteria can tolerate pH values between 2.9 and 9.5, their optimum growth is generally between pH 6–8 [100-102].

In addition to the decrease in CO_2_ and increase in CH_4_, we observed a decrease in N_2_O emissions in the +ASW treatments. N_2_O emissions in anaerobic sediments are primarily driven by denitrification rates, which, in turn, are driven by carbon (electron donor) availability, nitrate (electron acceptor) availability, and oxygen availability (high oxygen inhibits denitrification) [103]. The decrease in N_2_O emissions is in line with previous work [104-106] and may be because of low salt tolerance among the denitrifying taxa in this freshwater system. Abundances of the key denitrification gene *nirS* (nitrite reductase) decreased with salt addition in a field study in a tidal freshwater marsh, and denitrifier community composition was significantly affected by the increased salinity [104]. In our data set, *nirS* abundance did not change among the treatments, but the other nitrate reductase gene *nirD* was significantly lower in +ASW and + ASW-SO4 treatments (Fig. 6).

Our experiment yielded several surprising results including an increase in CH_4_ but decrease in CO_2_ and N_2_O emissions following ASW additions, a primary effect of non-sulfate seawater ions on microbial taxonomy and function, and a lack of an effect of sulfate on sulfate reducer populations, sulfate reduction gene abundances, and greenhouse gas emissions. We posit that the effects of NaCl likely drove the microbial responses and outweighed any effects of sulfate, but we cannot rule out any other potential effects of the other seawater components, which is an avenue for future research. Our field site is notable in that it is a forested freshwater wetland with a much lower pH (< 5.5) than most other studied tidal freshwater marshes. More research is needed on the effects of non-sulfate seawater ions on microbial communities and biogeochemistry, as well as how site and edaphic factors lead to discrepancies in seawater effects on greenhouse gas emissions. Cations may also travel farther inland than sulfate, and their impacts may thus cover larger areas during saltwater intrusion events [46]. Since our experiment was performed in the laboratory setting, we also suggest more research involving field manipulations [107] to test the effects of salinization while avoiding any laboratory artifacts. Such work is crucial for predicting the biogeochemical responses to the slowly but constantly rising sea levels on Earth.

## Supplementary Material

ISMEcomm_SI_Revised_ycae040

## Data Availability

16S OTU representative sequences are available on NCBI GenBank (PRJNA1005166). Metagenomic data are available on IMG/M and GOLD (GOLD study ID Gs0114296). All data and code to reproduce the results and figures is available via Zenodo https://zenodo.org/doi/10.5281/zenodo.10011195.
