# Supplementary material for: Disentangling the effects of sulfate and other seawater ions on microbial communities and greenhouse gas emissions in a coastal forested wetland
Source: ISME Commun. 2024 Mar 26;4(1):ycae040. doi: 10.1093/ismeco/ycae040 (PMC11020224; doi:10.1093/ismeco/ycae040)
Supplement: ISMEcomm_SI_Revised_ycae040 [file ismecomm_si_revised_ycae040.pdf]

Supplementary information for:

Disentangling the effects of sulfate and other seawater ions on microbial communities and greenhouse gas emissions in a coastal forested wetland

Clifton P. Bueno de Mesquita[1], Wyatt H. Hartman[1], Marcelo Ardón[2], Susannah G. Tringe[1,3,*]

[1] Department of Energy Joint Genome Institute, Lawrence Berkeley National Laboratory, Berkeley, CA 94720, USA

[2] Department of Forestry and Environmental Resources, North Carolina State University, Raleigh, NC 27695, USA

[3] Environmental Genomics and Systems Biology Division, Lawrence Berkeley National Laboratory, Berkeley, CA 94720, USA

*Corresponding author sgtringe@lbl.gov

Running title: Non-sulfate seawater ions affect microbes and fluxes

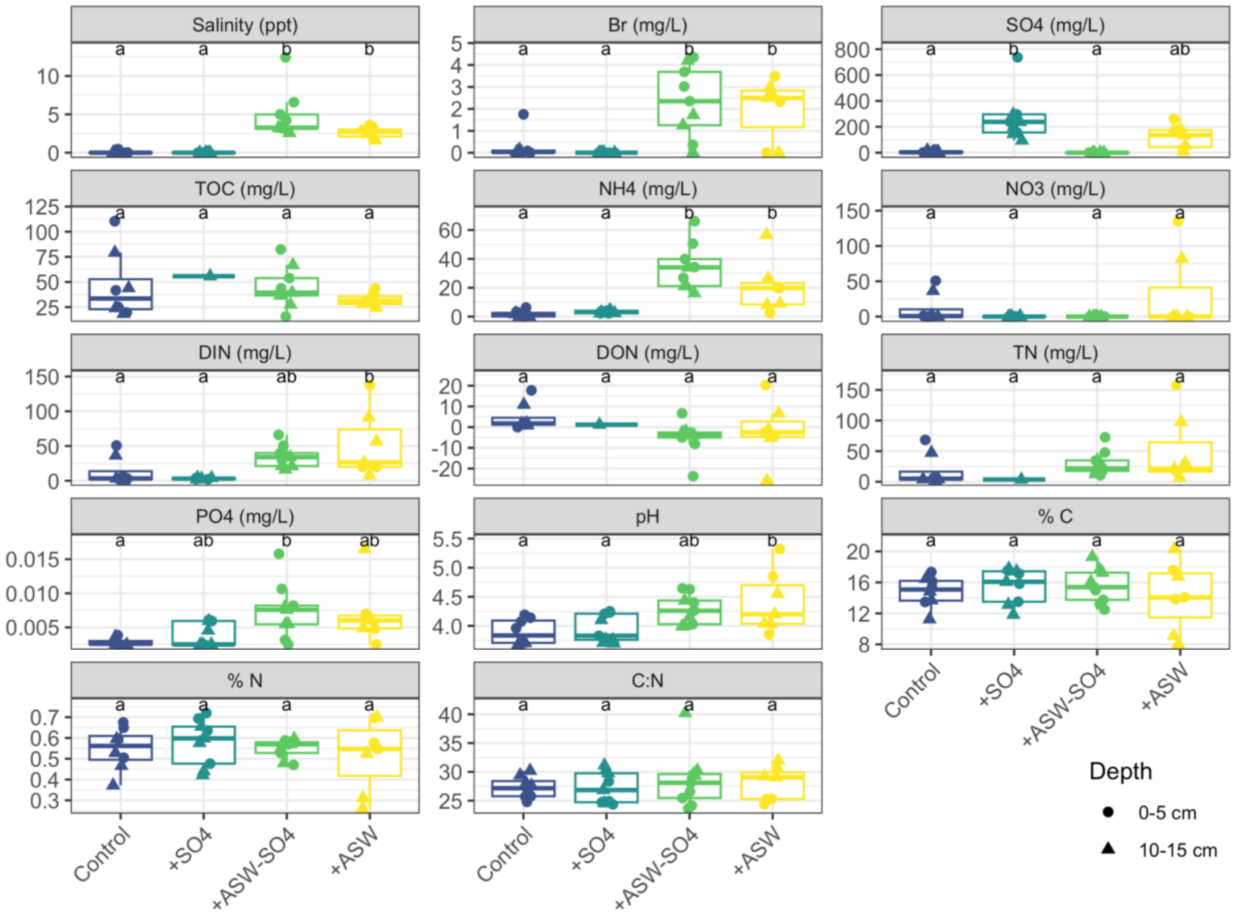

Figure S1. Biogeochemical data across the laboratory treatments. Different letters represent significant differences.

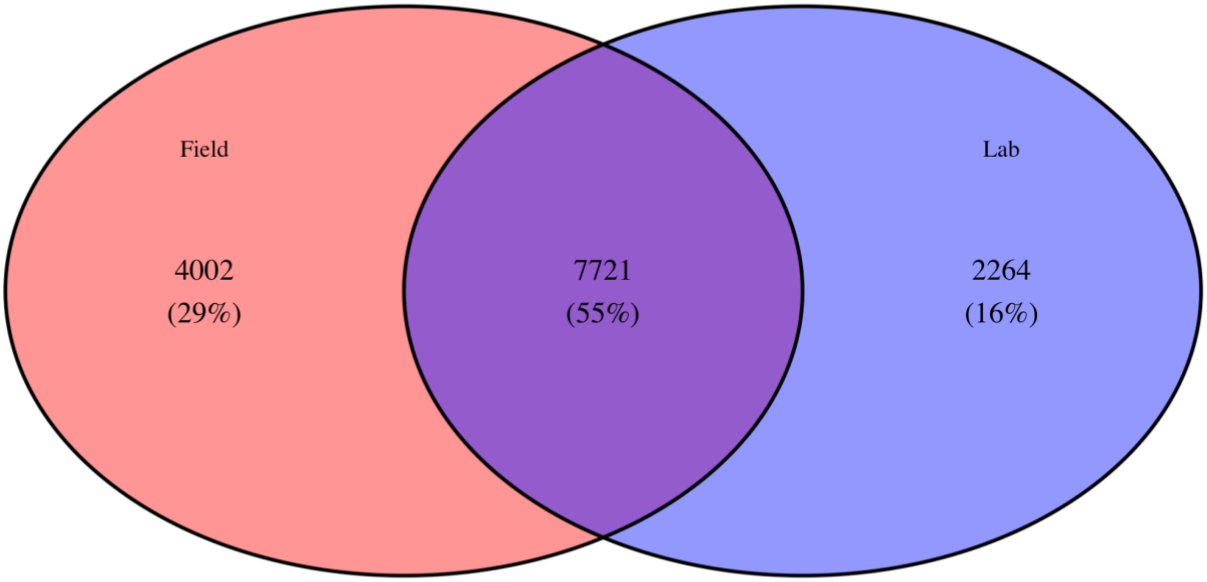

Figure S2. Venn diagram of the number of OTUs in field versus laboratory samples from 16S rRNA gene amplicon sequencing data.

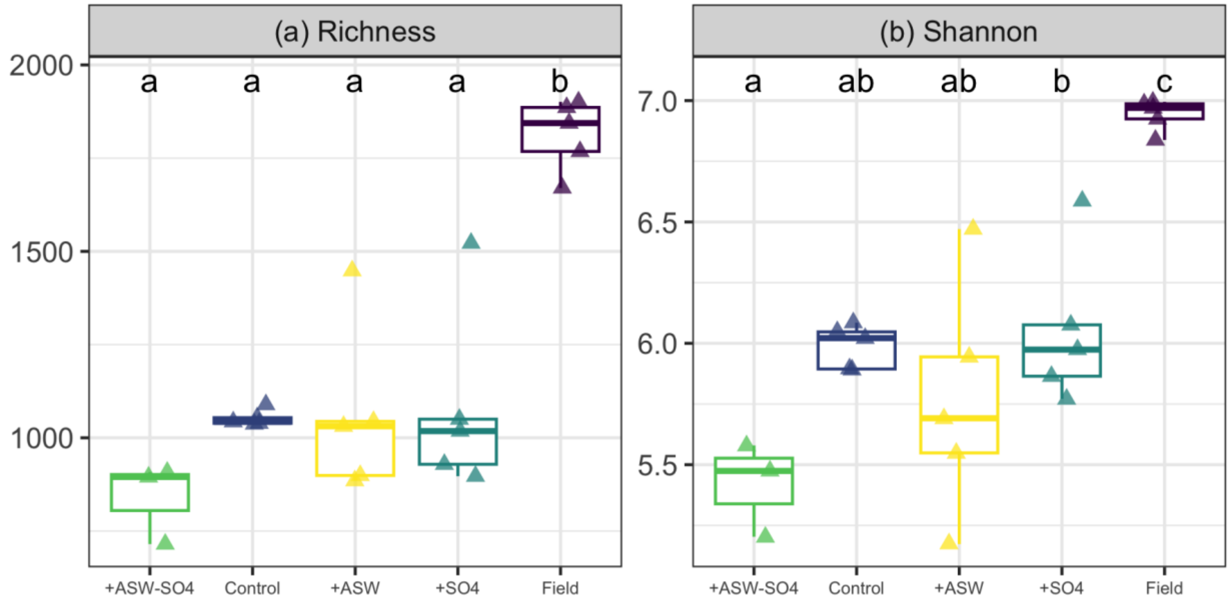

Figure S3. Alpha diversity of 10-15 cm depth samples according to mTAGs (16S genes extracted from metagenomes) showing (a) OTU richness, and (b) Shannon diversity. Note that only the 10-15 cm depth portion was sequenced for metagenomes and thus the 0-5 cm depth portion is not shown here. The x-axis is ordered by increasing mean richness.

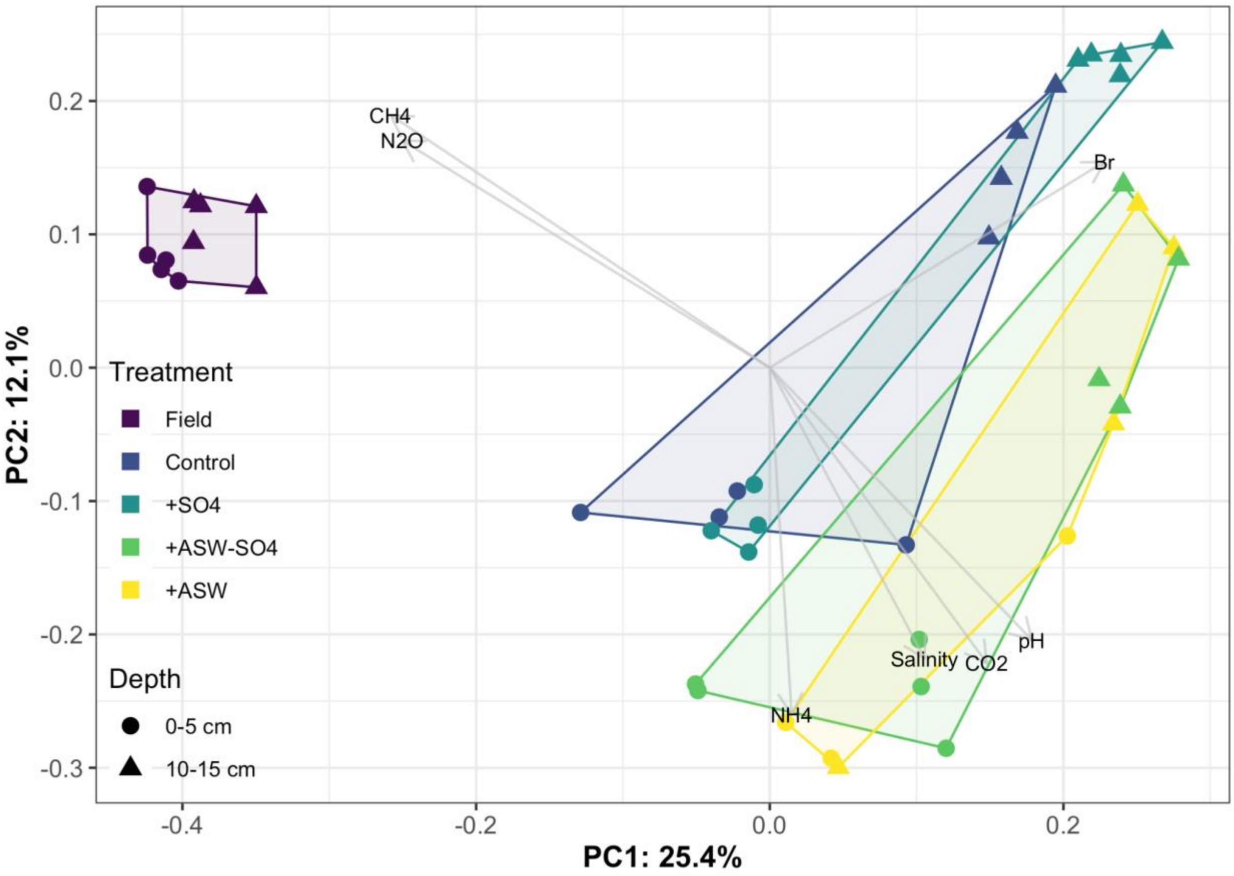

Figure S4. Principal coordinates analysis of Jaccard dissimilarity of 16S rRNA gene amplicon sequencing profiles. Vectors show environmental relationships with composition as calculated by ‘envfit’. Note that CO_2 and N_2O were not measured in field samples and those vectors represent higher values in controls and +SO4 samples.

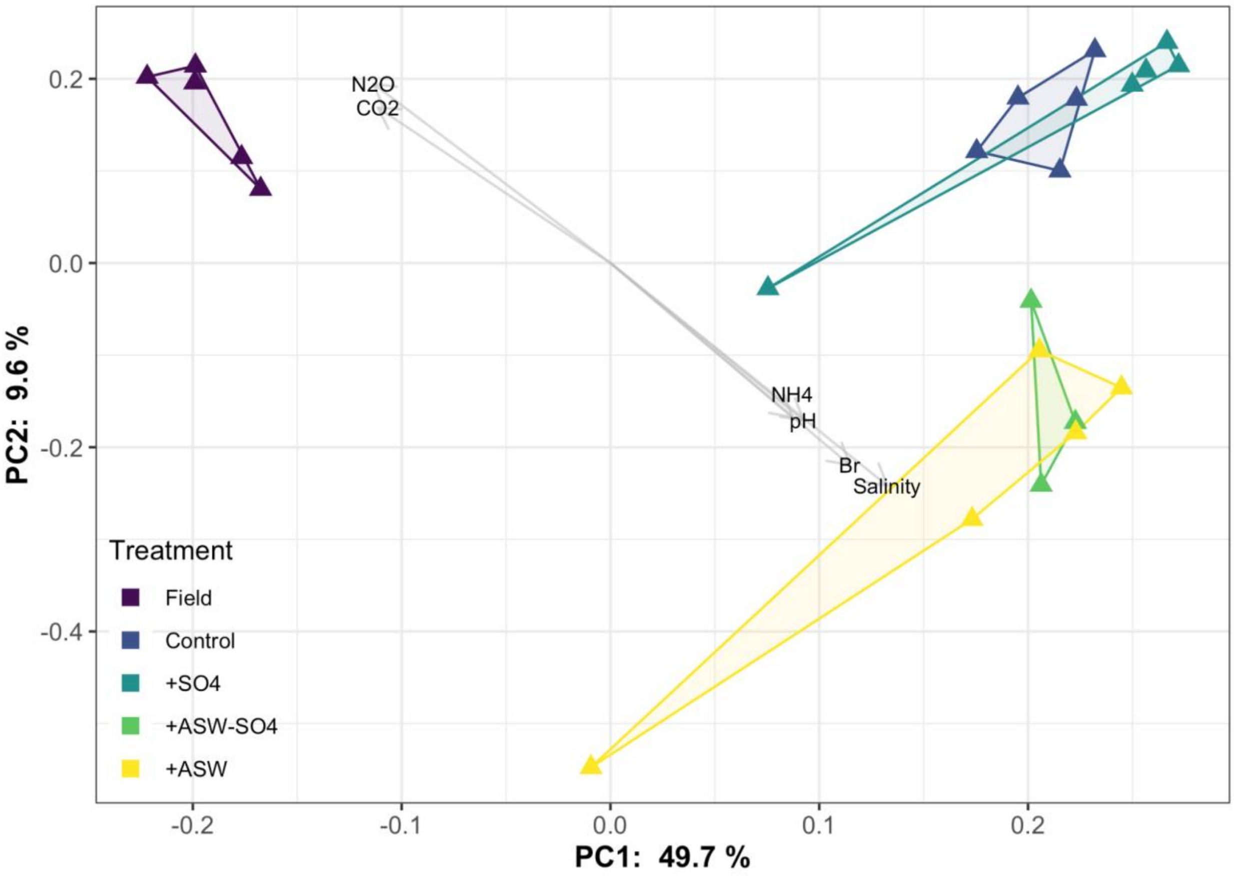

Figure S5. Principal components analysis of Aitchison distance for mTAGs (16S rRNA genes extracted from metagenomes). Vectors show environmental relationships with composition as calculated by 'envfit'. Note that only the 10-15 cm depth portion was sequenced for metagenomes and thus the 0-5 cm depth portion is not shown here.

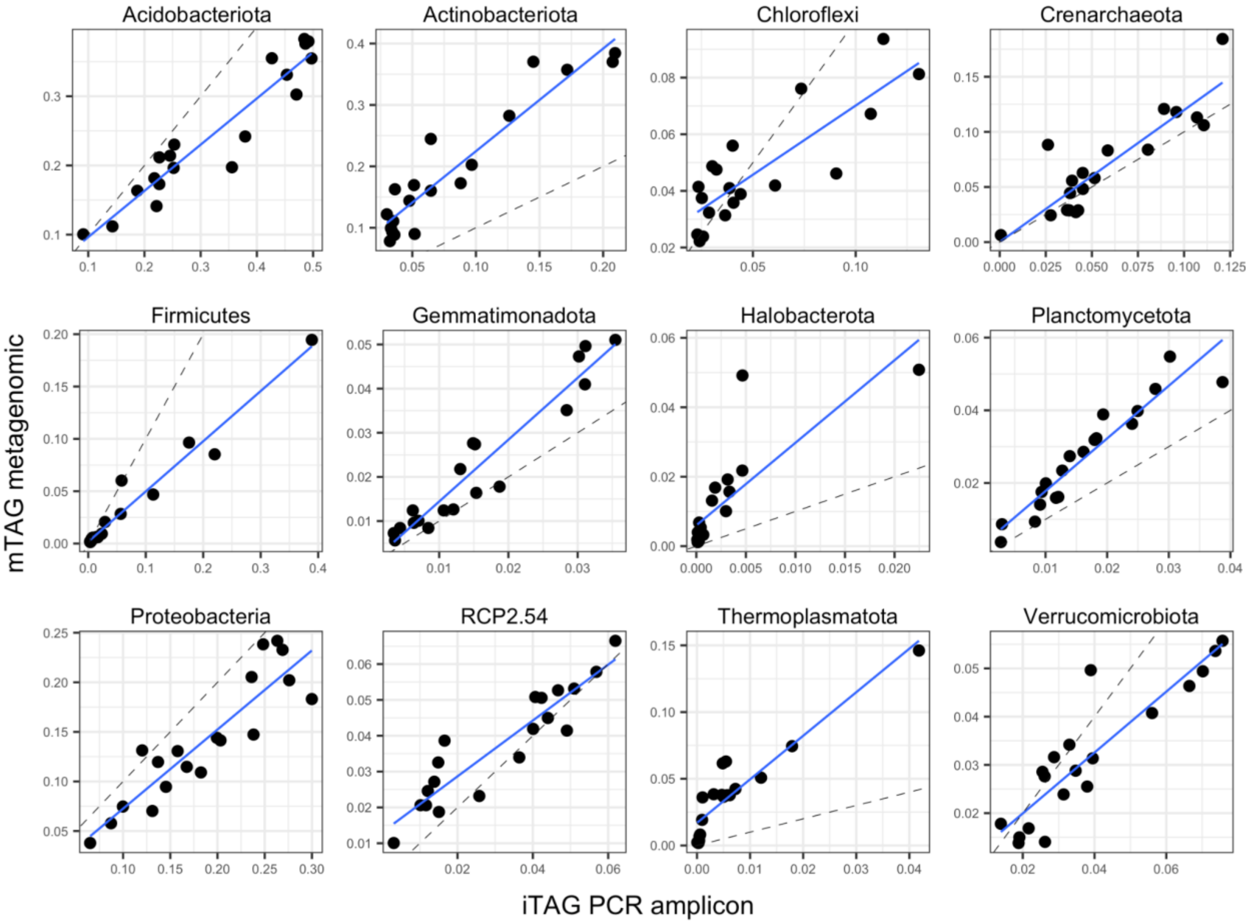

Figure S6. Comparison of iTAG (16S rRNA gene amplicon sequencing) vs. mTAG (16S rRNA genes extracted from metagenomes) abundances of the top 12 phyla for 5-15 cm depth samples that were sequenced for both 16S amplicons and shotgun metagenomics (n = 23). Blue lines represent linear regression lines while dashed lines represent the 1:1 line. Points to the right of the dashed line indicate greater abundances in the iTAG data while points to the left of the dashed line indicate greater abundances in the mTAG data.

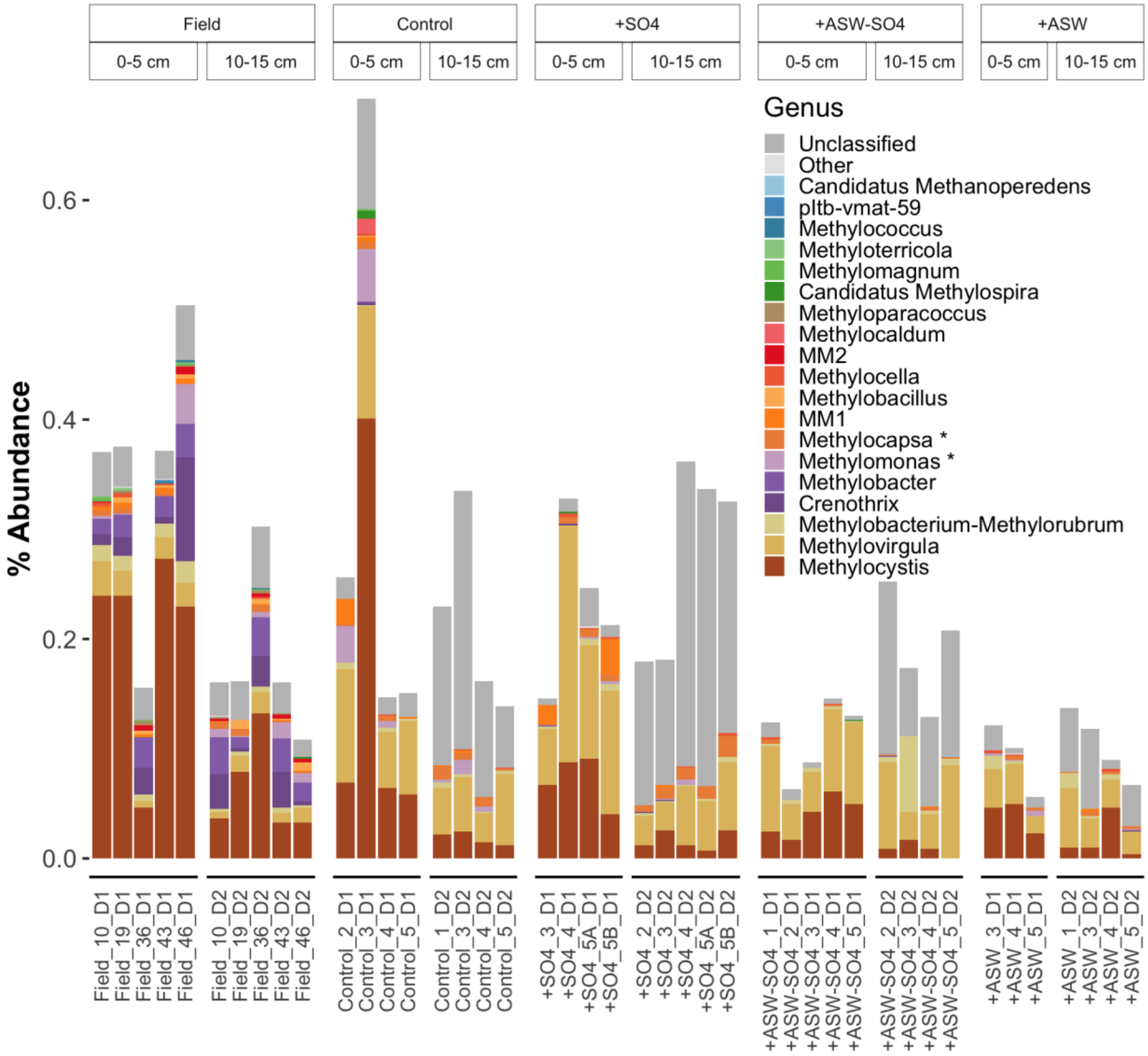

Figure S7. Methanotroph abundances from 16S rRNA gene amplicon sequencing, showing the top 19 most abundant methanotrophic genera, all other methanotrophic genera (“Other”), and unclassified methanotrophs at the genus level. *significant effect of treatment on abundance.

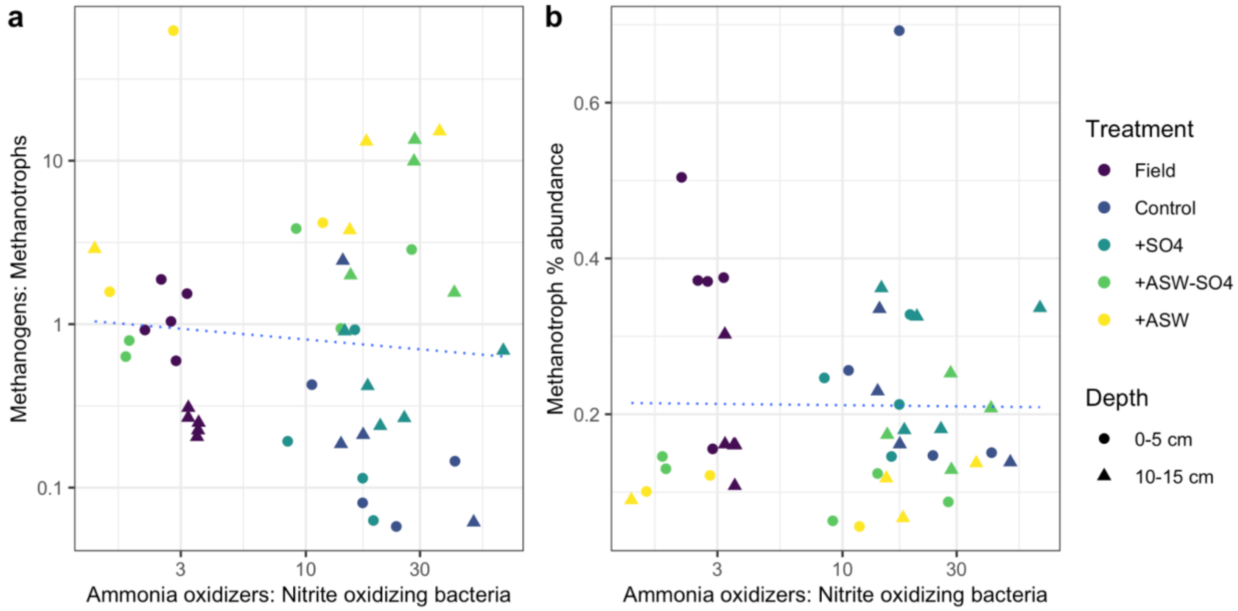

Figure S8. Lack of significant relationships between the ratio of ammonia oxidizers to nitrite oxidizing bacteria and (a) methanogen: methanotroph ratio, and (b) methanotroph percent relative abundance. Data from 16S rRNA gene amplicon sequencing data.

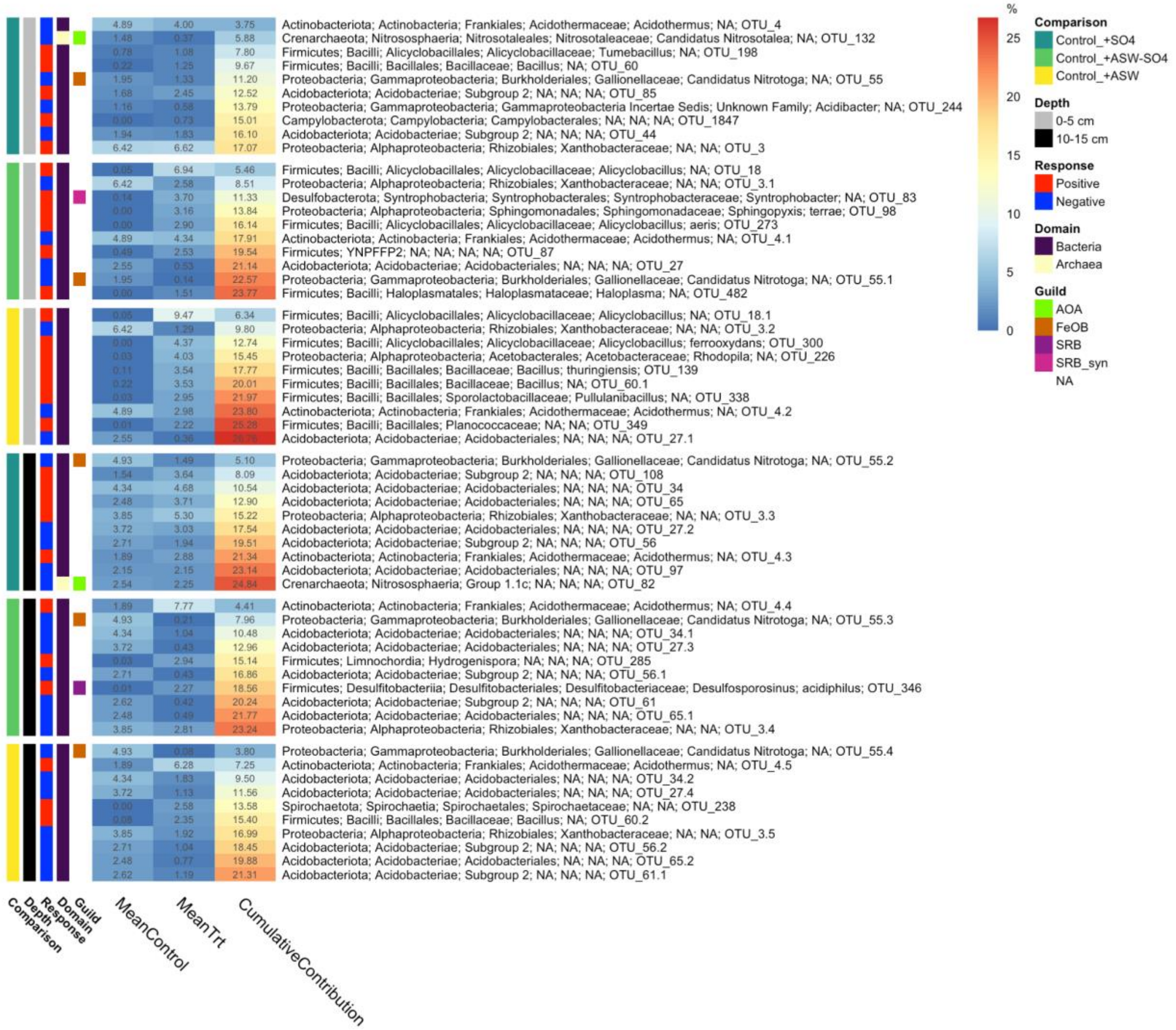

Figure S9. Results of SIMPER analysis performed on 16S rRNA gene amplicon sequencing data, showing the taxonomy top 10 OTUs contributing to dissimilarity in pairwise comparisons between each treatment and controls. OTUs numbers with decimal points indicate OTUs that are repeated in this figure (i.e., in the top 10 contributors to multiple pairwise comparisons).

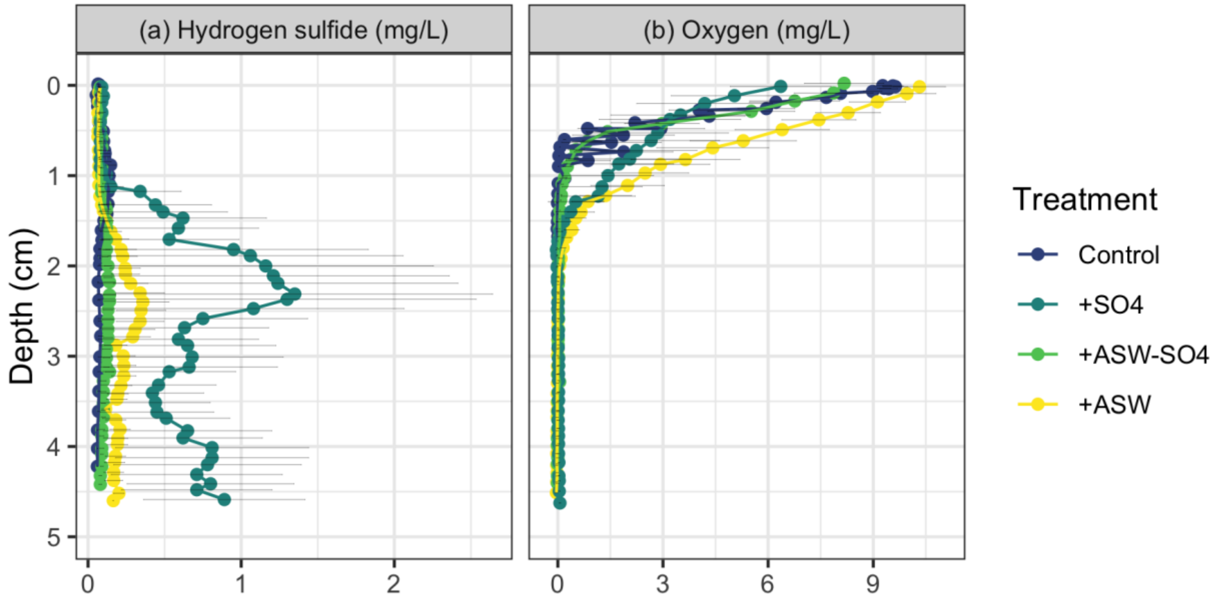

Figure S10. Mean (±SE) hydrogen sulfide (a) and oxygen (b) concentrations in the top 5 cm of water, measured at day 14 of the experiment. Note the difference in x-axis scale between the panels.
